# Bilateral Endogenous Endophthalmitis Secondary to Streptococcus pneumoniae: An Uncommon but Devastating Complication

**DOI:** 10.7759/cureus.37655

**Published:** 2023-04-16

**Authors:** Wen Jun Chua, Anuradha P. Radhakrishnan, Widad M Yusof, Chee Yik Chang

**Affiliations:** 1 Internal Medicine, Hospital Selayang, Selayang, MYS; 2 Infectious Diseases, Hospital Selayang, Selayang, MYS; 3 Ophthalmology, Hospital Selayang, Selayang, MYS

**Keywords:** streptococcus pneumoniae, hypopyon, vitrectomy, endogenous endophthalmitis, pneumococcal

## Abstract

Endophthalmitis is an infection of the vitreous and/or aqueous humours, caused by bacteria or fungi, and can be either exogenous (resulting from trauma or intraocular procedures) or endogenous (hematogenous in origin). Although less common than exogenous endophthalmitis, endogenous endophthalmitis can have serious, vision-threatening consequences. *Streptococcus pneumoniae* is a rare cause of endogenous endophthalmitis and is associated with a poor prognosis. In this report, we present a rare case of pneumococcal endogenous endophthalmitis that led to a devastating outcome despite both medical and surgical interventions. Early systemic treatment and prompt identification of the primary source are crucial and potentially life-saving.

## Introduction

Endophthalmitis is a serious infection that can result from the bacterial or fungal invasion of the vitreous and/or aqueous humours. There are two types of endophthalmitis: exogenous and endogenous. Exogenous endophthalmitis is more common and typically results from external factors such as corneal ulcers, trauma, or post-operative complications. In contrast, endogenous endophthalmitis is rare but can have devastating consequences due to the hematogenous spread of microorganisms from a distant site of infection [1, 2].

The causative agents of endogenous endophthalmitis can vary depending on the location, with *Streptococci sp.* being a primary cause in North America and Europe, and Gram-negative organisms such as *Klebsiella pneumoniae* being more common in Asia [2]. A local study found that *Klebsiella pneumoniae* was the most common pathogen causing endogenous endophthalmitis (32.5%), with *Streptococcus sp.* accounting for only 3.6% of cases [3]. Additionally, melioidosis is a rare infectious disease that can present with periorbital cellulitis, eyelid abscess, and endophthalmitis in endemic areas [4, 5].

Treatment options for endogenous endophthalmitis include intravitreal and systemic antibiotics with good ocular penetration, as well as vitrectomy. However, despite these interventions, visual outcomes remain poor in many cases [2, 3]. In this report, we present a case of bilateral endogenous endophthalmitis resulting from *Streptococcus pneumoniae* bacteraemia with significant ophthalmic sequalae, highlighting the severity of this condition and the need for early and aggressive management. Early systemic treatment and prompt identification of the primary source are crucial and potentially life-saving.

## Case presentation

A 60-year-old male retiree presented with a 5-day history of fever, multiple joint pains in both shoulders and the right knee, and acute bilateral blurring of vision with floaters that quickly deteriorated to only seeing shadows in two days. There was no history of nuchal rigidity, photophobia, or headache, ruling out meningitis. The patient had a history of immune thrombocytopenic purpura and was taking oral prednisolone 7.5 mg daily.

He initially presented to a private hospital in stable condition with normal vital signs. Physical examination revealed no abnormalities in the lungs, cardiovascular system, or neurological system. A joint examination, however, revealed swelling over the left shoulder and right knee. Joint aspiration was performed, but no fluid was obtained, indicating a dry tap. During an ophthalmic examination, it was discovered that the patient had bilateral hypopyon, as well as reduced visual acuity to hand movement in both eyes. A B-scan of both eyes revealed no obvious abnormalities.

The patient underwent a vitreous tap of both eyes and received empirical intravitreal injections of vancomycin (1 mg) and ceftazidime (2.5 mg) in both eyes while awaiting the culture results. The vitreous culture was negative but his blood culture revealed penicillin-sensitive *Streptococcus pneumoniae*, which was identified by the MALDI-TOF (Matrix-Assisted Laser Desorption/Ionization-Time of Flight). As a result, he was diagnosed with *S. pneumoniae* bacteremia with bilateral endogenous endophthalmitis.

**Figure 1:**
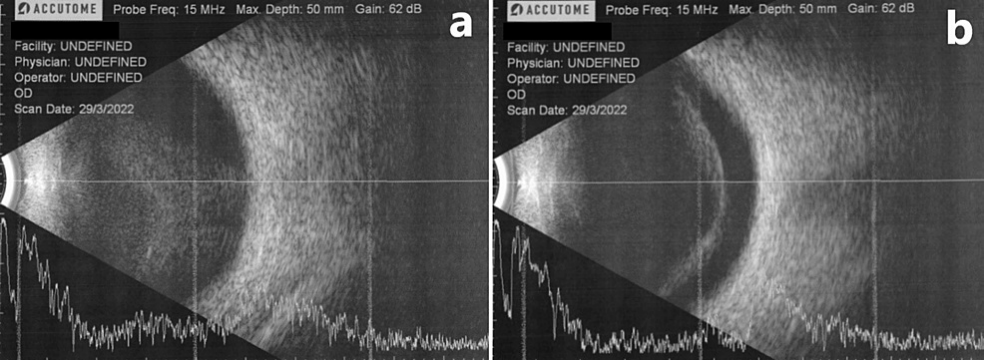
B-scan of the right eye revealed (a) vitreous opacities and (b) posterior vitreous detachment

Following the results, he was started on intravenous benzylpenicillin 4MU every 6 hours. A transthoracic echocardiogram revealed normal cardiac function and no vegetation, while a chest radiograph revealed no signs of pneumonia. Following a month of intravenous benzylpenicillin, the patient's left eye's visual acuity improved to 6/120, while his right eye's visual acuity initially improved to counting fingers 3 feet. As a result, the decision was made to switch from intravenous to oral penicillin. Unfortunately, his right-eye vision quickly deteriorated to light perception over the next few days. A B-scan revealed vitreous opacities and a posterior vitreous detachment (Figure 1).

The patient then requested to be discharged from the private hospital and transferred to our facility for a second opinion. Assessment done at our facility revealed visual acuities of light perception in his right eye and hand movement in his left eye, as well as intraocular pressures (IOP) of 4 mmHg and 12 mmHg in his right and left eyes, respectively. White conjunctiva, clear cornea, deep anterior chamber, occasional cells, iris pigment on the lens with nuclear sclerosis, and retrolental opacity were found in both eyes, with no fibrin or hypopyon. Conservative management was chosen for the right eye because of the poor prognosis (only light perception) and hypotonus (IOP < 5mmHg).

**Figure 2:**
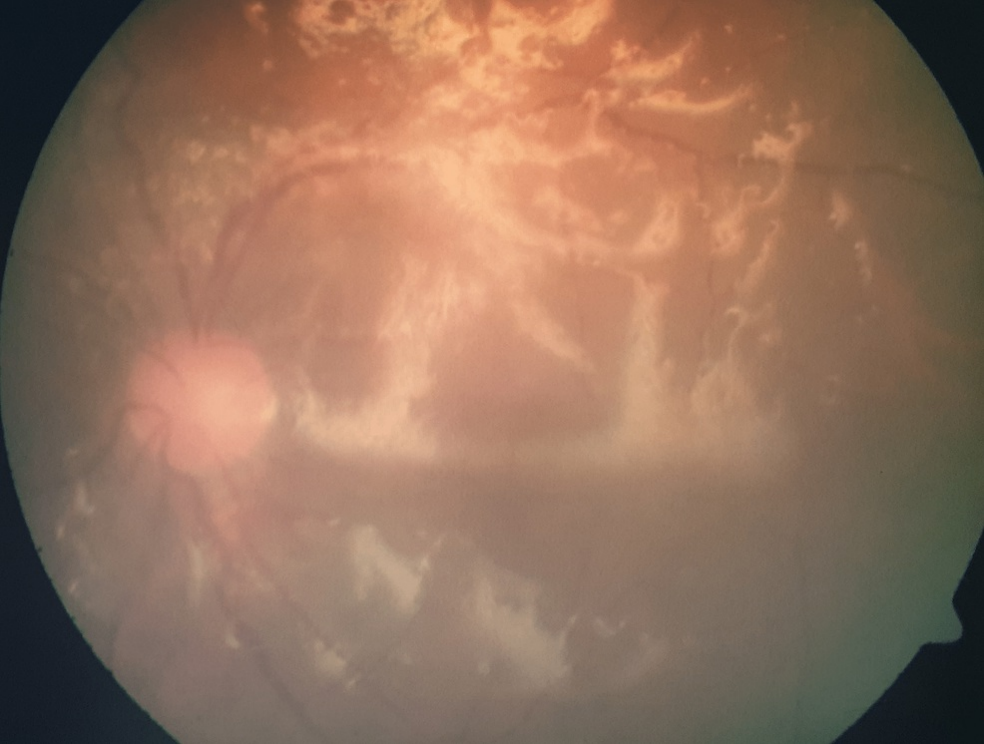
A fundoscopic examination of the left eye revealed that the disc is mildly hyperemic and that the vessels are tortuous; the glistening appearance is caused by the reflection of photographic flash by the oil/retina interface

The patient underwent vitreous sampling and vitrectomy on the left eye, as well as intravitreal antibiotics administered concurrently. The vitreous sample was negative for Gram stain and culture. A fundoscopic examination of the left eye two weeks after vitrectomy and silicone oil injection revealed a mildly hyperaemic disc and the vessels appeared tortuous (Figure 2). A slit lamp examination by the ophthalmology team revealed a normal macula with no retinitis or vasculitis.

While no organisms grew in vitreous and subsequent blood cultures, indicating that the intraocular infection had been successfully controlled, the patient was transitioned to intravenous ceftriaxone 2 g once daily for six weeks, with coordination between the ophthalmology and infectious diseases teams. The patient was discharged with light perception in his right eye and 6/36 in his left eye.

The patient's right eye visual acuity remained at light perception during a follow-up examination at the ophthalmology clinic, while the left eye had deteriorated to 6/60 due to cataract progression. The patient underwent left eye phacoemulsification, intraocular lens implantation, and silicone oil removal six months later. During the most recent ophthalmology clinic visit, the right eye's visual acuity was hand movement (right eye intraocular pressure = 6 mmHg), and the left eye's visual acuity was 6/24 (left eye intraocular pressure = 12 mmHg).

## Discussion

Endophthalmitis, a severe inflammatory condition of the eye, is associated with significant destruction of the retina due to its limited regenerative capacity. The blood-retina barrier, a vital component of ocular immunity, normally prevents the infiltration of pathogens and non-ocular immune cells into the retina. However, in endophthalmitis, this barrier becomes dysfunctional, allowing for the direct infiltration of pathogens and non-ocular immune cells, leading to ocular tissue damage [6]. Exogenous endophthalmitis is the most common type of endophthalmitis, accounting for about 80% of cases, while endogenous endophthalmitis accounts for approximately 20% of cases [7].

Endogenous endophthalmitis can be caused by various microorganisms depending on geographical location and patient-specific risk factors. Risk factors for endogenous endophthalmitis include underlying chronic illnesses such as diabetes, renal insufficiency, and malignancy, as well as immunocompromised patients with leukemia, lymphoma, asplenia, or hypogammaglobulinemia, and those taking immunosuppressive therapy, such as corticosteroids [3,8]. Here, we present a rare case of pneumococcal endogenous endophthalmitis, which is an unusual manifestation of the infection, particularly in the Southeast Asia region. The case also highlights the importance of coordinated care between the ophthalmology and infectious diseases teams, as the patient's management required a combination of systemic antibiotics, intra-vitreal injections of antibiotics, and surgery.

Unilateral endogenous endophthalmitis is typically treated with a combination of antibiotics and surgical intervention to minimize the risk of ocular invasion in the unaffected eye and to preserve vision. However, because systemically administered antibiotics have limited ability to penetrate the blood-ocular barrier, systemic and intravitreal antibiotics are typically used to treat distant foci of infection in endogenous endophthalmitis [9]. Early diagnosis and antibiotic administration (within 24 hours of diagnosis) have been shown to result in a favorable visual prognosis [10]. Nevertheless, endogenous endophthalmitis caused by *Streptococcus pneumoniae* is generally associated with poor visual outcomes, despite the use of appropriate sensitive antibiotics [3].

Vitrectomy is a valuable tool in the diagnosis and treatment of severe endophthalmitis when systemic and intravitreal antibiotics are not effective. The Endophthalmitis Vitrectomy Study Group (EVS) evaluated the effectiveness of immediate vitrectomy and intravenous antibiotics for postoperative bacterial endophthalmitis. The study found that performing an immediate vitrectomy did not result in significant differences in visual outcomes compared to not performing one, except in cases where patients had initial light perception vision where immediate vitrectomy showed significant benefits. Additionally, the timing of vitrectomy did not significantly impact visual prognosis, with no difference seen between early vitrectomy (within two weeks) and delayed vitrectomy (more than two weeks) [11].

Endogenous endophthalmitis is a potentially sight-threatening condition that can have variable outcomes depending on the causative organism. While gram-positive organisms are the most commonly isolated, fungal infections tend to have the worst outcomes. In the East Asian population, the literature review shows that about 34% of cases had a visual acuity of counting fingers or better, while about 16% required evisceration or enucleation of the eye [12]. The emergence of hypervirulent strains of *Klebsiella pneumoniae* has led to an increased incidence of endophthalmitis in Southeast Asia, which tends to be caused by gram-negative organisms [13]. Among these, *Streptococcus pneumoniae* carries a worse prognosis when compared to *Klebsiella pneumoniae* and *Pseudomonas aeruginosa* [3,14].

## Conclusions

In conclusion, endogenous endophthalmitis is a rare but serious ocular infection with a poor visual prognosis, and it must be managed as an ophthalmic emergency. In our presented case, bilateral endogenous endophthalmitis was caused by *Streptococcus pneumoniae* bacteraemia, which is a rare occurrence. Early recognition, prompt management with intravitreal and systemic antibiotics, and vitrectomy are essential for the preservation of visual acuity in patients with endogenous endophthalmitis. However, despite these interventions, visual outcomes remain poor in many cases. Therefore, patients require adequate follow-up and supportive care from a multidisciplinary team of healthcare professionals, including ophthalmologists, infectious disease specialists, and primary care physicians, to minimize the impact of visual impairment on their quality of life.
